# High-level amplification at 17q23 leads to coordinated overexpression of multiple adjacent genes in breast cancer

**DOI:** 10.1038/sj.bjc.6603692

**Published:** 2007-03-13

**Authors:** J Pärssinen, T Kuukasjärvi, R Karhu, A Kallioniemi

**Affiliations:** 1 Laboratory of Cancer Genetics, Institute of Medical Technology, Tampere University Hospital and University of Tampere, University of Tampere, Tampere, FIN-33014, Finland; 2 Department of Pathology, Tampere University Hospital, Tampere, FIN-33520, Finland

**Keywords:** breast cancer, gene amplification, gene expression

## Abstract

Increased copy numbers of 17q23 chromosomal region have been shown to occur in different tumour types and to be associated with tumour progression and with poor prognosis. Several genes have earlier been proposed as potential oncogenes at this region largely on the grounds of cell lines studies. In this study, we performed a systematic gene expression survey on 26 primary breast tumours with known 17q23 amplification status by quantitative real-time reverse transcriptase-polymerase chain reaction (RT-PCR). The 17q23 amplicon is restricted to an ∼5 MB region in breast cancer and contains 29 known genes. Our survey revealed a statistically significant (*P*<0.01) difference between the high level and no amplification groups in a set of eleven genes whereas no difference between the moderate and the non-amplified tumour groups were observed. Interestingly, these 11 genes were located adjacent to one another within a 1.56 Mb core region in which all except one of the genes were overexpressed. These data suggest that only high-level amplification at the 17q23 amplicon core leads to elevated gene expression in breast cancer. Moreover, our results highlight the fact that 17q23 amplicon carries multiple candidate genes and that this may be a more common event in gene amplification than previously thought.

Gene amplification is one of the major mechanisms that allow cancer cells to promote expression of genes that are involved in tumour development and progression. Oncogene overexpression as a result of gene amplification has been shown to play a crucial part in the pathogenesis of various human malignancies, especially in solid tumours such as breast, prostate, lung, ovarian, gastric, pancreatic, and colon cancers (Knuutila *et al*, 1998). The first illustration on the clinical significance of amplified oncogenes in human cancer was the discovery of an association between amplification of the *MYC* oncogene and more aggressive neuroblastoma variants (Schwab, 1993). To date, one of the clinically most important amplified gene is the *ERBB2* oncogene, which is well known for its central role as a prognostic and predictive factor in breast cancer as well as a therapeutic target (Baselga *et al*, 1999; Cobleigh *et al*, 1999; Ross and Fletcher, 1999; Vogel *et al*, 2002). These examples illustrate that genes that are altered by amplification in cancer are likely to have an impact on both disease pathogenesis and the clinical management of cancer patients.

Amplification of the chromosomal region 17q23 was first discovered in breast cancer (Kallioniemi *et al*, 1994). After that, numerous studies have reported increased copy numbers of 17q23 in tumours of brain, lung, ovary, pancreas, bladder, testis, and liver (Muleris *et al*, 1994; Ried *et al*, 1994; Korn *et al*, 1996; Solinas-Toldo *et al*, 1996; Brinkschmidt *et al*, 1997; Marchio *et al*, 1997; Richter *et al*, 1997; Schwendel *et al*, 1997; Sonoda *et al*, 1997; Weber *et al*, 1997; Vandesompele *et al*, 1998; Clark *et al*, 2002; Willis *et al*, 2003). We recently performed an extensive survey on the distribution and frequency of the 17q23 copy number increases in 3520 tumours representing 166 different tumour types (Andersen *et al*, 2002). The results confirmed previous data and indicated that increased 17q23 copy number occurs most commonly in brain, lung, breast, ovarian, urinary bladder, and soft tissue tumours, although high-level amplification was observed exclusively in breast cancer. Importantly, the increased copy number of 17q23 region has also been associated with tumour progression (Andersen *et al*, 2002) and with poor prognosis in breast cancer (Isola *et al*, 1995; Barlund *et al*, 2000a), ovarian clear cell adenocarcinoma (Hirasawa *et al*, 2003), neuroblastoma (Saito-Ohara *et al*, 2003), and acute myelogenous leukaemia (Morerio *et al*, 2001). Taken together, these data suggest that the genes affected by the 17q23 amplification contribute to cancer pathogenesis.

In breast cancer, several studies have been performed to define the limits of the 17q23 amplicon (Barlund *et al*, 1997, 2000b; Couch *et al*, 1999; Erson *et al*, 2001; Monni *et al*, 2001; Wu *et al*, 2001). Based on the combination of data from these studies and the current information available in the human genome databases (http://www.ncbi.nlm.nih.gov/mapview/ and http://www.ensembl.org/Homo_sapiens/index.html), the amplicon is considerably large covering an approximately 5 Mb region at 17q23. A number of studies have also aimed to uncover the possible target genes for this amplification, mainly by looking at correlation between amplification and high-level mRNA expression. One of the first genes to be identified as a potential oncogene in this region was the ribosomal protein S6 kinase, *RPS6KB1* (Couch *et al*, 1999; Barlund *et al*, 2000a). Thereafter several other putative target genes have been proposed including *APPBP2* (also known as *PAT1*), *RAD51C*, *TBX2*, *TRIM37* (*MUL*), *THRAP1* (*TRAP240*), *PPM1D*, and *BRIP1* (Barlund *et al*, 2000b; Wu *et al*, 2000, 2001; Erson *et al*, 2001; Monni *et al*, 2001; Bulavin *et al*, 2002; Li *et al*, 2002). However, these studies were carried out at a time when the genome sequence of this region was incomplete and thus all genes within this region had not been identified. In addition, these previous studies mainly concentrated on the analysis of established breast cancer cell lines and the expression levels of only five genes from this region have been evaluated in a small number of primary breast tumours (Couch *et al*, 1999; Barlund *et al*, 2000b; Li *et al*, 2002).

In the present study, we aimed to further characterise the molecular consequences of the 17q23 amplification on gene expression levels in primary breast tumours. To this end, all known genes localised within the 5 Mb amplicon at 17q23 were obtained from publicly available databases and their expression levels were measured in 26 primary breast tumours using quantitative real-time reverse transcriptase-polymerase chain reaction (qRT-PCR). Our data revealed that high-level amplification at 17q23 in primary breast cancer leads to coordinated overexpression of 11 adjacent genes located at a 1.56 Mb central region of the amplicon.

## MATERIALS AND METHODS

### Primary tumours and breast cancer cell lines

Freshly frozen primary breast tumour specimens and corresponding paraffin-embedded tissue samples from 26 breast cancer patients were acquired from the Department of Pathology, Tampere University Hospital. These samples represent a subset of a larger material that has been described in detail previously (Rauta *et al*, 2006). The clinicopathological characteristics of these 26 tumour samples are presented in Table 1. The mean age of the patients at diagnosis was 62 years (range 38–84). The patients had not received any therapy before tumour removal. The use of these specimens in this study was approved by the Ethics Committee of the Pirkanmaa Hospital District and by the National Authority for Medicolegal Affairs in Finland.

Two breast cancer cell lines BT-474 and MCF7 were included in this study. Pancreatic cancer cell lines PANC-1 and HUPT3 as well as prostate cancer cell lines DU145 and PC-3 were used as controls in RT-PCR analyses. HUPT3 cell line came from the German Collection of Micro-organisms and Cell Cultures (Braunschweig, Germany), all other cell lines were obtained from the American Type Culture Collection (ATCC, Manassas, VA, USA). Cell lines were cultured under recommended conditions. Normal testis and pancreas cDNA (Ambion, Foster City, CA, USA) were also used as controls in RT-PCR analyses. Human Mammary Gland (HMG) cDNA was from BD Biosciences Clontech (Palo Alto, CA, USA).

### Copy number analysis by fluorescence *in situ* hybridisation

Three pairs of bacterial artificial chromosome (BAC) clones corresponding to the 17q23 chromosome region (probe set A: RP11-579A4 and RP11-579O24; probe set B: RP11-634F5 and RP11-1081E4; probe set C: RP11-269G24 and CTD-2501B8) were identified from public databases (http://www.ncbi.nlm.nih.gov/mapview/ and http://www.ensembl.org). BAC DNA was labelled with SpectrumOrange-dUTP (Vysis, Downers Grove, IL, USA) by random priming and a SpectrumGreen labelled chromosome 17 probe (Vysis) was used as a reference. Fluorescence *in situ* hybridisation (FISH) to normal metaphase chromosomes was performed to confirm that the clone contigs recognised a single copy target at 17q23. Fluorescence *in situ* hybridisation on paraffin-embedded breast tumour samples was performed using a modified Paraffin Pretreatment Reagent kit protocol (Vysis) as described previously (Rauta *et al*, 2006). Hybridisation signals were analysed using an Olympus BX50 epifluorescence microscope (Tokyo, Japan) using an × 60 objective (NA 1.4). Specimens containing a three to fivefold increase in the number of gene specific probe signals, as compared with the chromosome 17 centromere signals, were considered to be moderately amplified. In addition, specimens containing a fivefold or higher increase in the number of *PPM1D* signals or tight clusters of signals were considered to be highly amplified. The results using the RP11-634F5 and RP11-1081E4 BAC contigs were included in a previous publication (Rauta *et al*, 2006).

### RNA isolation and cDNA synthesis

Total RNA was extracted from cancer cell lines using TRIZOL Reagent (Invitrogen, Carlsbad, CA, USA) according to manufacturer's instructions. For primary tumour samples, a representative tumour area was selected from freshly frozen tumour specimen based on haematoxylin–eosin stained tissue section and a core-biopsy (∅2 mm) was obtained for RNA isolation. Tumour tissues were homogenised with a syringe and a needle (20G, ∅0.9 mm) and total RNA was extracted with RNeasy Mini Kit (Qiagen, Valencia, CA, USA). First-strand cDNA synthesis for both cell line and primary tumour RNAs was performed using SuperScript II reverse transcriptase and random hexamer primers (Invitrogen).

### Expression screen by RT-PCR

Gene specific primers for 29 genes from the 17q23 region (primer sequences are available on request) were obtained from TIB MolBiol (Berlin, Germany). The PCR contained 1 × PCR Gold buffer (Applied Biosystems, Foster city, CA, USA), 1.5 mM MgCl_2_, 0.2 mM dNTPs each, 0.2–0.4 mM gene specific primers, 1.5–2.5 units of Amplitaq Gold DNA polymerase (Applied Biosystems), and 1 *μ*l cDNA template (MCF7 or BT474) adjusted to 50 *μ*l with sterile H_2_O. The PCR programme began with initial denaturation at 95°C for 5 min, followed by 35 cycles of denaturation at 95°C for 30 s, annealing at 55°C for 60 s and elongation at 72°C for 60 s, with final elongation at 72°C for 10 min. The PCR products were run on a 1.5% agarose gel. For those genes with no detectable expression in MCF7 and BT-474 cells, the functionality of the PCR primers was verified using cDNA from PANC-1, HUPT3, DU145, PC-3, normal testis or normal pancreas as a template.

### Quantitative real-time RT-PCR

DNA Hybridisation Probe Sets for 24 genes at 17q23 and the housekeeping gene TATA-box-binding protein (*TBP*) were obtained from TIB MolBiol (Berlin, Germany). The PCR reactions were performed in the LightCycler apparatus using the LC FastStart DNA Hybridisation Probes Kit according to manufacturer's instructions (Roche Diagnostics, Mannheim, Germany). Briefly, the PCR contained 1 × LightCycler FastStart DNA Master HybProbe mix (Roche Applied Science, Mannheim, Germany), 4 mM MgCl_2_, 0.2–0.4 *μ*M gene specific probes, 0.8 *μ*M gene specific primers, and 1.5 *μ*l cDNA template adjusted to 20 *μ*l with sterile H_2_O. After 10 min of initial denaturation at 95°C, the cycling conditions (55 cycles) were as follows: denaturation at 95°C for 10 s, annealing at 55–58°C for 10–20 s, and elongation at 72°C for 6–12 s depending on the gene of interest. Quantitative analysis was performed using the LightCycler software according to the fit-point method as described earlier (Kauraniemi *et al*, 2003). The expression levels were normalised against the housekeeping gene *TBP*.

### Statistical analysis

For each gene, two-tailed Mann–Whitney *U*-test was used to examine the possible statistical significance of differences in expression levels between the three tumour groups. Three separate comparisons (high *vs* moderate, high *vs* no, and moderate *vs* no amplification tumour group) were made.

## RESULTS

### Primary tumour selection and copy number analysis

We had previously determined the DNA copy number levels within the centre of the 17q23 amplicon (at the *PPM1D* gene locus) in a set of 146 primary breast tumours by using formalin-fixed, paraffin-embedded samples (Rauta *et al*, 2006). On the basis of these data, a total of 26 tumours, including eight cases with increased copy number and 18 tumours with no copy number increase, were selected for this study owing to the availability of freshly frozen tumour material required for the mRNA expression analyses. To determine further the 17q23 amplification status in this set of samples, we used additional probe pairs from both ends of the 5 Mb amplicon and determined their copy number levels by FISH (Table 2). As might be expected, the 18 non-amplified tumours did not show copy number increase with the additional probe sets either (data not shown). The eight amplified cases demonstrated more or less consistent copy number changes across the entire amplicon and could now be classified into groups with high (*n*=4) and moderate (*n*=4) level of amplification (Table 2).

### Transcript identification

**Figure 1:**
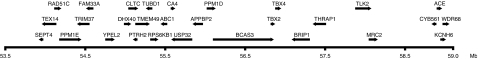
Physical map of the 17q23 amplicon. The known genes mapping to the ∼5 Mb minimal region of amplification at 17q23 are represented using horizontal lines and their orientation is indicated with arrowheads.

On the basis of previous studies (Barlund *et al*, 1997, 2000b; Couch *et al*, 1999; Erson *et al*, 2001; Monni *et al*, 2001; Wu *et al*, 2001) and the current genome sequence information (http://www.ncbi.nlm.nih.gov/mapview/ and http://www.ensembl.org/Homo_sapiens/index.html), the 17q23 amplicon is restricted to an ∼5 Mb region (53.95–59.02 Mb) and contains 29 genes with known function (Figure 1, Table 3). First, the expression levels of these 29 genes were screened by regular RT-PCR in MCF7 and BT-474 breast cancer cell lines that are known to harbour high level 17q23 amplification (Couch *et al*, 1999; Barlund *et al*, 2000a; Monni *et al*, 2001). Five (*SEPT4, TEX14, CA4, MRC2,* and *KCNH6*) of the 29 genes had either very low or undetectable expression in both MCF7 and BT-474 cells (data not shown) and were thus excluded from further analyses based on the fact that a potential amplification target gene is expected to show elevated mRNA expression.

### Expression screen in primary breast tumours

**Figure 2:**
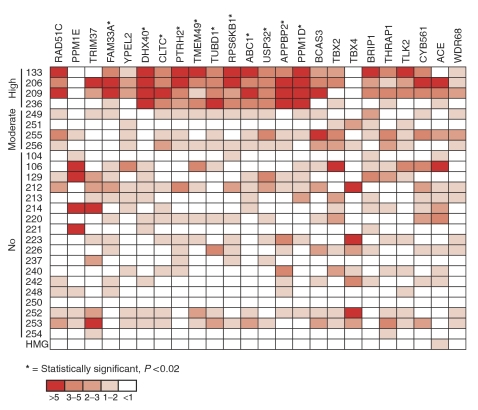
Schematic representation of expression levels of 24 known genes within the 17q23 chromosomal region in 26 primary breast tumours and normal HMG. Primary tumours are arranged according to their 17q23 amplification status and genes are organised based on their physical order at the 17q23 region from centromere to telomere. Expression levels were determined using qRT-PCR and were normalised against a housekeeping gene *TBP*. The relative expression values of each gene were median-corrected and displayed using a colour code (shown at the bottom).

The expression levels of the remaining 24 genes from the 17q23 amplicon were then measured in the 26 primary breast tumours using qRT-PCR. Two genes, *PTRH2* and *ACE*, showed considerably higher expression levels than any other gene within this region (Table 3). However, *ACE* was also expressed at a similar level in normal HMG, and therefore does not appear to be interesting as putative target gene for 17q23 amplification. The expression levels of *PPM1E* were very low across all 26 tumour samples and, additionally, both *PPM1E* and *TBX4* showed highest expression levels in non-amplified tumour samples (Table 3, Figure 2).

**Figure 3:**
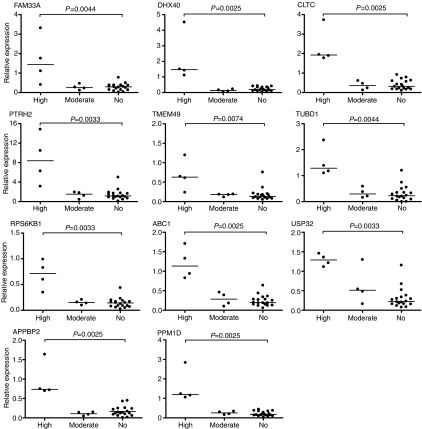
Comparison of *FAM33A, DHX40, CLTC, PTRH2, TMEM49, TUBD1, RPS6KB1, ABC1, USP32, APPBP2,* and *PPM1D* expression in highly, moderately, and no amplification tumour groups by qRT-PCR. Median value of expression is indicated by a horizontal line. Two-tailed Mann–Whitney *U*-test was used to assess the statistical significance of differences in expression levels between tumour groups. *P*-values between highly non-amplified groups are shown.

Finally, the expression levels of the 24 genes within the 17q23 amplicon were compared between the three tumour groups. A statistically significant (*P*<0.01) difference between the high level and no amplification groups were observed for a set of 11 genes (*FAM33A*, *DHX40*, *CLTC*, *PTRH2*, *TMEM49*, *TUBD1*, *RPS6KB1*, *ABC1*, *USP32*, *APPBP2*, and *PPM1D*) (Figure 3). All of these, except *FAM33A* and *USP32*, also showed significant difference (*P*<0.05) in expression levels between the high-and moderate-level amplification groups suggesting that these genes are activated by high-level copy number increases (Figure 3). No difference between the moderate and the no amplification tumour groups were observed. Interestingly, these 11 genes are located adjacent to each other within a 1.56 Mb region (54.54–56.10 Mb) at the centre of the 17q23 amplicon (Figures 1 and 2). It was even more intriguing that only a single gene, *YPEL2*, within this region did not demonstrate an association between amplification and increased mRNA expression.

## DISCUSSION

Several studies have illustrated recurrent amplification of 17q23 in various human tumours and its association to poor clinical outcome (Isola *et al*, 1995; Barlund *et al*, 2000a; Morerio *et al*, 2001; Andersen *et al*, 2002; Hirasawa *et al*, 2003; Saito-Ohara *et al*, 2003). These studies have proposed multiple genes from this region to be important for cancer development and progression and thus promising targets for diagnostic, prognostic, and therapeutic approaches. However, no systematic survey of all genes within the 17q23 region has earlier been performed in primary tumour material. Here 29 known genes located within the common amplified segment at 17q23 were defined. Five of these were excluded from further analysis, because they showed very low or undetectable expression in cell lines with high level amplification. The expression levels of the remaining 24 genes were then examined using qRT-PCR in 26 primary breast tumours to assess their role as putative target genes for 17q23 amplification in breast cancer.

Our systematic study revealed eleven genes (*FAM33A*, *DHX40*, *CLTC*, *PTRH2*, *TMEM49*, *TUBD1*, *RPS6KB1*, *ABC1*, *USP32*, *APPBP2*, *PPM1D*) that showed significantly (*P*<0.01) higher expression levels in primary breast tumours with high level 17q23 amplification compared to tumours without amplification. Interestingly, these eleven genes are all located within a 1.56 Mb region at the centre of the 17q23 amplicon. The amplicon core also includes one additional gene, *YPEL2*, but for unknown reasons it does not demonstrate copy number dependent overexpression. As only very little is known about the function of this gene, it is not possible to speculate on the basis of this phenomenon. Overall, our findings are in good agreement with previous studies that have implicated gene copy number alterations as significant determinants of gene expression patterns (Hyman *et al*, 2002; Pollack *et al*, 2002; Wolf *et al*, 2004; Fridlyand *et al*, 2006). However, it has to be noted that the tumour group with moderate amplification showed expression levels similar to those seen in the non-amplified tumours, indicating that low level copy number increases at this region did not have a significant effect on gene expression levels. This finding is supported by recent studies demonstrating that the high-level amplifications but not copy number gains are associated with poor prognosis in various tumour types (Sticht *et al*, 2005; Staebler *et al*, 2006). In summary, the qRT-PCR screen demonstrated that high level amplification at 17q23 in breast cancer leads to concomitant overexpression of virtually all genes within the amplicon core.

Several genes have earlier been proposed as putative target genes of the 17q23 amplification. These include *RPS6KB1*, *APPBP2,* and *PPM1D* (Couch *et al*, 1999; Barlund *et al*, 2000a, 2000b; Wu *et al*, 2001; Bulavin *et al*, 2002; Li *et al*, 2002) that also showed strong association between amplification and increased expression in our primary tumour material. Earlier studies have also implicated *RAD51C, TRIM37, TBX2, THRAP1*, and *BRIP1* as genes whose expression correlates with amplification (Barlund *et al*, 2000b; Wu *et al*, 2000, 2001; Erson *et al*, 2001; Monni *et al*, 2001). In our tumour series, these genes did also show elevated expression in some samples with high level amplification, but the difference in expression levels between the high and no amplification tumour groups, did not reach statistical significance. This finding might reflect the fact that we focussed solely on the analysis of primary tumour material instead of established breast cancer cell lines that were mainly used in these previous studies. Finally, our data revealed seven other protein coding genes (*FAM33A*, *DHX40*, *CLTC*, *PTRH2*, *TMEM49*, *TUBD1*, *ABC1*, and *USP32*) that have not been implicated previously in 17q23 studies, but whose expression was clearly associated with copy number status in primary breast tumours. Taken together, our results highlight a set of new genes that are overexpressed because of the 17q23 amplification in breast cancer and thus might contribute to the tumour phenotype.

Similar observations on overexpression of multiple genes within an amplicon have been reported previously in several occasions. For example, recent studies identified a 1 Mb segment of common amplification at 8p11–12 in breast cancer and demonstrated that it contains at least 14 candidate genes showing a strong correlation between amplification and overexpression (Garcia *et al*, 2005; Gelsi-Boyer *et al*, 2005). At 11q13, which is amplified in a wide variety of tumours (Saunders *et al*, 2000; Gollin, 2001, 2004), Huang *et al* (2006) constructed a detailed 3.6 Mb map of the amplicon core and showed that most of the genes in that region are overexpressed in amplified tumours and cell lines. Similarly, we have demonstrated that amplification of a 280 kb minimal common region at 17q12 in breast cancer leads to simultaneous increase of expression levels of all genes within that genomic segment (Kauraniemi *et al*, 2001). In general, these data implicate that despite the size of the affected region, the vast majority of the genes within amplicons are overexpressed and hence amplification events typically lead to overexpression of multiple genes. Owing to this tight association between amplification and elevated gene expression, it is difficult to determine which of the genes within particular amplicon actually contribute to tumour pathogenesis. It is likely that all of the overexpressed genes are not critical for cancer progression but are overexpressed merely because of their location within the amplicon. However, it is also possible that, instead of a single target gene, amplicons are driven by a set of genes whose simultaneous overexpression provides growth advantage to cancer cells (Huang *et al*, 2006; Kao and Pollack, 2006).

Traditionally, the search for amplification target genes has been based on two primary schemes; namely location within the maximal amplitude of the amplicon and overexpression of the putative target gene. Our results demonstrate that a whole set of genes, 11 of the 12 known genes within the 1.56 Mb core region, is overexpressed in primary breast tumours with high level amplification at 17q23. Owing to such coordinated overexpression of genes in the amplification core, overexpression alone is not sufficient to highlight putative target genes but functional studies are required to uncover the possible biological and clinical significance of candidate genes.

## Figures and Tables

**Table 1:** Clinicopathological characteristics of 26 primary breast tumours

**Variable**	***n***	**%**
*Tumour type*		
Infiltrating ductal	22	84.6
Infiltrating lobular	4	15.4
		
*Tumour size*		
<20 mm	12	46.2
⩾20 mm	14	53.8
		
*Histological grade* a		
I	0	0.0
II	8	36.4
III	14	63.6
		
*Stage*		
T1	11	42.3
T2	5	19.2
T3	4	15.4
T4	6	23.1
		
*Nodal status*		
Negative	12	46.2
Positive	11	42.3
Not available	3	11.5
		
*ER*		
Positive	21	81.8
Negative	5	19.2
		
*PR*		
Positive	13	50.0
Negative	13	50.0
		
*ERBB2*		
Negative	21	80.8
Positive	5	19.2

a Histological grade was determined only from the infiltrating ductal carcinomas.

**Table 2:** Copy number levels at the 17q23 amplicon relative to chromosome 17 centromere in eight primary breast tumours by FISH

	**Probe sets^a^**		
**Tumour number**	**A^b^**	**B^c^**	**C^d^**
*High*			
133	1.5	Amp^e^	3
206	Amp^e^	Amp^e^	2.5
209	Amp^e^	Amp^e^	Amp^e^
236	1	>5	1
			
*Moderate*			
249	1.5	3.5	1
251	3	3.5	3.5
255	2	3	2
256	3.5	3.5	3

FISH=fluorescence *in situ* hybridisation.

a See Materials and Methods.

b Includes *RAD51C-PPM1E genes*.

c Includes *PPM1D gene*.

d Includes *CYB561-ACE-KCNH6-WDR68* genes.

e Tight cluster of signals indicative of amplification.

**Table 3:** List of the 29 known genes located in the 5 Mb minimal region of amplification at 17q23 and their minimum, maximum and median expression levels in 26 primary breast tumours

**Gene**	**Description**	**Min**	**Max**	**Median**
SEPT4	Peanut-like protein 2	ND	ND	ND
TEX14	Testis expressed sequence 14	ND	ND	ND
RAD51C	DNA repair protein RAD51 homolog	0.0	1.9	0.3
PPM1E	Protein phosphatase 1E	0.0	1.7	0.0
TRIM37	Tripartite motif-containing 37 protein	0.0	1.1	0.2
FAM33A	Family with sequence similarity 33, member A	0.0	3.3	0.3
YPEL2	Yippee-like 2 protein	0.1	1.0	0.3
DHX40	DEAH (Asp-Glu-Ala-His) box polypeptide 40	0.0	4.5	0.2
CLTC	Clathrin heavy chain 1	0.0	3.7	0.4
PTRH2	Peptidyl-tRNA hydrolase 2	0.1	14.8	1.4
TMEM49	Transmembrane protein 49	0.0	1.2	0.2
TUBD1	Tubulin δ chain	0.0	2.4	0.3
RPS6KB1	Ribosomal protein S6 kinase	0.0	1.0	0.2
ABC1	Amplified in breast cancer	0.0	1.7	0.3
CA4	Carbonic anhydrase IV precursor	ND	ND	ND
USP32	Ubiquitin C-terminal hydrolase 32	0.0	1.5	0.3
APPBP2	Amyloid β precursor protein binding protein 2	0.0	1.6	0.1
PPM1D	Protein phosphatase 2C δ isoform, magnesium-dependent	0.0	2.8	0.2
BCAS3	Breast carcinoma amplified sequence 3	0.1	2.7	0.3
TBX2	T-box transcription factor TBX2	0.0	0.7	0.1
TBX4	T-box transcription factor TBX4	0.0	9.0	0.3
BRIP1	BRCA1 interacting protein C-terminal helicase 1	0.0	2.1	0.2
THRAP1	Thyroid hormone receptor-associated protein	0.0	1.1	0.2
TLK2	Serine/threonine-protein kinase tousled-like 2	0.1	1.6	0.3
MRC2	Mannose receptor, C type 2	ND	ND	ND
CYB561	Cytochrome b561	0.3	9.2	1.3
ACE	Angiotensin-converting enzyme, somatic isoform precursor	4.0	233.0	32.1
KCNH6	Potassium voltage-gated channel, subfamily H, member 6	ND	ND	ND
WRD68	WD-repeat protein 68	0.1	1.8	0.8

ND=not determined (low or now expression in breast cancer cell lines MCF7 and BT-474 with high-level amplification).
